# Clinical effectiveness of suture anchor repair combined with open reduction and internal fixation in the treatment of deltoid ligament rupture in ankle fracture

**DOI:** 10.12669/pjms.39.6.8070

**Published:** 2023

**Authors:** Le Zhang, Xiaochuan Kong, Gang Hong, Yinfeng Zheng, Lei Zang

**Affiliations:** 1 Le Zhang, Department of Orthopedics, Beijing Chao-Yang Hospital, Capital Medical University, Beijing 100043, P.R. China; 2 Xiaochuan Kong, Department of Orthopedics, Beijing Chao-Yang Hospital, Capital Medical University, Beijing 100043, P.R. China; 3 Gang Hong, Department of Orthopedics, Beijing Chao-Yang Hospital, Capital Medical University, Beijing 100043, P.R. China; 4 Yinfeng Zheng, Department of Orthopedics, Beijing Chao-Yang Hospital, Capital Medical University, Beijing 100043, P.R. China; 5 Lei Zang, Department of Orthopedics, Beijing Chao-Yang Hospital, Capital Medical University, Beijing 100043, P.R. China

**Keywords:** Ankle fracture, Deltoid ligament rupture, Open reduction and internal fixation, Suture anchor repair

## Abstract

**Objective:**

To explore the clinical effectiveness of suture anchor (SA) repair combined with open reduction and internal fixation (ORIF) in the treatment of deltoid ligament rupture (DLR) in ankle fractures.

**Methods:**

This is a retrospective analysis of 210 patients with DLR in ankle fracture who were treated in Beijing Chaoyang Hospital from January 2020 to June 2022. According to the surgical records, 125 patients received SA repair combined with ORIF (Repair group) and 85 patients received ORIF only (Non-repair group). The curative effect, recovery of ankle joint function, pain, and bone metabolism of the two groups were observed.

**Results:**

The clinical effectiveness (overall good) was higher in the Repair group (*P*<0.05). The American Orthopedic Foot and Ankle Society (AOFAS) score was higher three and six months post-operation in the Repair group, and the Visual Analogue Scale (VAS) score was lower than that of the Non-repair group (*P*<0.05). The Repair group had higher levels of bone-specific alkaline phosphatase (BALP) and bone gla protein (BGP) than the Non-repair group six months post-operation (*P*<0.05).

**Conclusions:**

SA combined with ORIF has a good effect in the treatment of DLR in ankle fracture patients, which can promote the recovery of ankle function, relieve postoperative pain and improve bone metabolism.

## INTRODUCTION

Patients with an ankle fracture experience ankle pain and swelling, directly impacting their mobility.^1^ Deltoid ligament rupture (DLR) is a common injury associated with this kind of fracture, and patients with DLR and an ankle fracture are usually treated with open reduction and internal fixation (ORIF).^1^-^3^ However, there is controversy about whether this surgical method can effectively repair the DLR.^4^ Some studies show that the DLR can repair itself over time without special surgical repair.^4^,^5^ However, other studies have found that if the DLR is not repaired in time, it may aggravate ankle pain and increase the risk of sequelae such as ankle instability.^5^,^6^

Traditional triangular ligament repair mainly includes deep insertion, direct suture, and steel wire bone tunnel suture.^7^ However, the traditional repair method is complicated and can cause trauma.^8^ In recent years, the application of suture anchor (SA) in the repair of ligament injury in fracture patients has become more and more common. This type of anchor has a tail line at the end, and adopts high and low threads in design, allowing the smooth implantation of the anchor into the patient’s bone tissue during the repair operation. SA is convenient to operate, can reduce injury, and is more conducive to promoting ligament healing and the functional recovery of ankle joints post-operation.^7^,^9^

Based on the above advantages of SA, we hypothesize that combined SA with ORIF may have a greater improvement in ankle function and decreased pain in patients with DLR in ankle fractures. Currently, there is little data on this combined approach. Therefore, this study reviewed and compared the clinical effectiveness, post-operative American Orthopedic Foot and Ankle Society (AOFAS), Visual Analogue Scale (VAS) scores and bone metabolism of patients with DLR in ankle fracture treated with SA repair combined with ORIF and simple ORIF, with the intention of providing a clinical reference.

## METHODS

A total of 210 patients with DLR in ankle fractures treated in Beijing Chaoyang Hospital from January 2020 to June 2022 were selected retrospectively, including 112 males and 98 females. The average age was 51.63 ± 13.33 years. Surgical records showed that 125 cases were treated with SA repair combined with ORIF (Repair group), while 85 cases were treated with ORIF only (non-repair group).

### Inclusion criteria:

- The results of CT imaging confirmed DLR in ankle fracture.^1^
- Age ≥ 18 years old.
- Patients with a unilateral fresh closed fracture.
- The function of the affected limb was good before the fracture.
- The clinical data was complete.

### Exclusion criteria:

- Those with functional diseases of the liver, kidney, heart, and other important organs.
- Patients with a history of ankle surgery.
- Those with pathological or open fractures.
- Those with obvious dysfunction of the ankle joint before the injury.
- Patients with malignant tumors.
- Patients with cognitive disorders, mental disorders, or mental diseases.
- Pregnant or lactating women.

### Ethical Approval

This study was approved by the medical ethics committee of our hospital (Ethics NO. 2023-Science-241; Date: 2023-03-15).

### Non-repair group

Patients in the Non-repair group received ORIF only. The surgical procedure of ORIF was as follows;^10^ A CT scan of the patients’ affected limb was performed before the operation to determine the severity of the ankle fracture. Basic treatments such as routine detumescence and support maintenance were performed according to the condition of the patient. The patient was placed in the recumbent position and the affected limb was raised. The skin and subcutaneous tissue of the patient’s lateral malleolus were cut layer by layer to expose the lateral malleolus, and the broken end of the lateral malleolus fracture was treated under direct vision. The anatomical locking plate (Smith & Nephew) was used to fix the patient’s lateral ankle. After the operation, the affected limb was supported and fixed with plaster. Ankle joint functional training was provided based on the patient’s recovery.

### Repair group

Patients in the Repair group received ORIF combined with SA. The surgical procedure of SA was as follows: A suture absorbable anchor Lupine (Johnson & Johnson) was selected for reconstruction and fixation of the medial triangular ligament. Superficial ligament injury was repaired directly. Deep ligament rupture and avulsion of the attachment point of the medial malleolus required the placement of the SA in the medial malleolus, and the triangular ligament was sutured and fixed. After the operation, the ankle joint was fixed with short leg plaster for six weeks, the patients were guided to carry out ankle joint functional exercise under ankle protection measures, and weight-bearing walking exercise was carried out 12 weeks after the operation.

### 1) Clinical effect evaluation:

The curative effect of patients was evaluated by the Baird-Jackson ankle joint scoring standard,^11^ with a score of 0 to 100, of which 0 to 80 points were poor, 81 to 90 points were average, 91 to 95 points were good, and 96 to 100 points were excellent. Total excellent and good rate = (number of cases with good curative effect + number of cases with excellent curative effect)/total number of cases × 100.0%.

2) Ankle function. Before, three- and six-months post-operation, the AOFAS scoring scale^12^ was used to assess the pain (40 points), functional and autonomous activities, support (10 points), maximum walking distance (five points), abnormal gait (eight points), ground walking (five points), anteroposterior activity (eight points), hind foot activity (six points), foot alignment (10 points) and ankle-posterior foot stability (eight points). The total score of the scale was 0 to 100 points, the higher the score, the better the ankle function.

3) Pain. Before, three- and six-months post-operation, VAS evaluation was carried out.^13^ The score was 0 to 10, 0 was painless, and 10 was maximum pain, the higher the score, the more severe the pain.

4) Bone metabolism. Before and six months post-operation, 5ml of the patient’s venous blood under fasting condition was collected and centrifuged at 4000rpm for eight minutes to obtain serum, and the serum BALP and BGP levels were measured with the enzyme-linked immunosorbent assay (ELISA) kit (manufacturer: Wuhan Elirit Biotechnology Co., Ltd.).

### Statistical analysis

Categorical variables were described as frequency and percentage (n, %), and differences between the groups were compared via Chi-squared tests. Continuous variables that do not comply with normal distribution were described as median (IQR), and differences between the two groups were compared using Mann-Whitney U-tests. *p* <0 .05 was the significance threshold. SPSS v25.0 (SPSS Inc., Chicago, IL, USA) was used for all statistical testing.

## RESULTS

This study included 210 patients with an ankle fracture, 125 in the Repair group and 85 in the Non-repair group. There were 69 males and 56 females in the Repair group and the average age was 52.74 ± 13.08 years. The causes of fracture within the Repair group were as follows: 68 cases of traffic accidents, 28 cases of falling from height, 20 cases of falling, nine cases of sprain. There were 43 males and 42 females in the Non-repair group and the average age was 50.01 ± 13.62 years. The causes of fracture within the Non-repair group were as follows: 45 cases of traffic accidents, 19 cases of falling from height, 14 cases of falling, seven cases of sprain (Table-I).

**Table-I:** Baseline characteristics.

Groups	Age, year	Gender, male/female	Cause of fracture, n (%)			

			Traffic accidents	Falling from height	Falling	Sprain
Repair group (n=125)	56.00(46.50-62.00)	69/56	68(54.40)	28(22.40)	20(16.00)	9(7.20)
Non-repair group (n=85)	52.00(41.50-61.00)	43/42	45(52.94)	19(22.35)	14(16.47)	7(8.24)
	-1.305	0.432	0.098			
*P*	0.192	0.511	0.992			

There was no significant difference in general data such as age, sex and fracture cause between the two groups (*P*>0.05). The total excellent and good results of treatment in the Repair group was 84.80%, significantly higher than 61.18% in the Non-repair group (*P*<0.05; Table-II). There was no significant difference in preoperative AOFAS score and VAS score between the two groups (*P*>0.05). The AOFAS score of the two groups increased and the VAS score decreased three- and six-months post-operation. The AOFAS score of the Repair group was significantly higher than that of the Non-repair group, and the VAS score was significantly lower than that of the Non-repair group (*P*<0.05; Table-III). There was no significant difference in the levels of BALP and BGP between the two groups pre-operation (*P*>0.05). The levels of BALP and BGP in the two groups increased six months post-operation and were significantly higher in the Repair group (*P*<0.05; Table-IV).

**Table-II:** Comparison of clinical efficacy between the two groups [n(%)].

	Excellent	Good	In general	Poor	Overall good and good rate
Repair group (n=125)	58 (46.40)	48 (38.40)	19 (15.20)	0 (0.00)	106 (84.80)
Non-repair group (n=85)	20 (23.53)	32 (37.65)	27 (31.76)	6 (7.06)	52 (61.18)
*Z*	4.622				15.156
*P*	<0.001				<0.001

**Table-III:** Comparison of AOFAS score and VAS score between the two groups (score, *χ̅*±*S*).

	AOFAS score			VAS score		
	
	Preoperative	Three months post-surgery	Six months post-surgery	Preoperative	Three months post-surgery	Six months post-surgery
Repair group (n=125)	53.00(47.50-56.00)	79.00(73.50-82.00)^a^	87.00(81.50-90.00)^a^	6.00(5.00-8.00)	2.00(2.00-3.00)^a^	1.00(1.00-2.00)^a^
Non-repair group (n=85)	52.00(44.00-56.00)	71.00(62.50-75.00)^a^	78.00(70.00-82.00)^a^	7.00(5.00-8.00)	3.00(2.00-4.50)^a^	2.00(1.00-3.00)^a^
*Z*	-0.975	-7.087	-7.701	-0.429	-3.226	-3.916
*P*	0.330	<0.001	<0.001	0.668	0.001	<0.001

***Note:*** Compared with preoperative results in this group,

a P<0.05.

**Table-IV:** Changes in bone metabolism (*χ̅*±*S*).

Group	BALP (U/L)			BGP (μg/L)

	Preoperative	Six months post-surgery	Preoperative	Six months post-surgery
Repair group (n=125)	82.00(71.50-90.50)	136.00(124.50-149.00)^a^	3.60(2.80-4.20)	7.40(6.50-8.45)^a^
Non-repair group (n=85)	85.00(73.00-93.00)	119.00(103.50-128.00)^a^	3.40(2.60-4.15)	5.80(5.10-6.85)^a^
*Z*	-1.028	-7.575	-1.167	6.959
*P*	0.304	<0.001	0.243	<0.001

***Note:*** Compared with preoperative results in this group,

a P<0.05.

## DISCUSSION

The results of this study showed that the treatment of DLR in ankle fracture with SA repair combined with ORIF is more clinically effective with greater improvement in ankle function and decreased pain, compared with simple ORIF. There is controversy regarding repairing the ligament following DLR in ankle fracture, with some studies showing that patients are prone to pain, swelling and stiffness post-ligament repair.^14^,^15^ Other studies recommend repairing the triangular ligament during the operation, to decrease ankle instability post-operation and improve healing.^16^,^17^ A systematic review^16^ containing nine studies (n=508) showed that deltoid ligament repair patients had the same or better results than the Non-repair group, in terms of pain, joint function, range of motion, medial clear space and incidence of complications. A meta-analysis of eight comparative studies (n=388) by Guo W *et al*^17^ found that patients who received DLR repair experienced improved medial clear space, AOFAS score and complication rate, with no significant difference in VAS score compared to patients without DLR repair.

Another systematic review of five studies (n=284) also showed that repairing the deltoid ligament was advantageous.^4^ Currently, it is preferred to use suture fixation and deep embedding of the insertion point to repair the triangular ligament, however, this operation is more complex, and may increase the risk of complications.^4^-^9^ The application of a suture anchor nail in the repair of the triangular ligament has been proven to be safe, can reduce soft tissue damage, and convenient, which can aid in maintaining joint function.^16^-^18^ Work by Luo G *et al*^9^ found that using a suture anchor to repair the deltoid ligament resulted in significant improvements. Additionally, in treatment of a high fibular fracture with combined ligament injury, the tightrope anchor was superior to screw fixation.^19^ Further, when comparing two kinds of non-absorbable materials used to suture the complete rupture of the deltoid ligament through bone drilling and suture anchor, it seems the two suture methods were both clinically effective in treating the complete rupture of the deltoid ligament, but the anchoring suture was difficult to remove.^7^ Further research is needed to determine more effective suture methods for this patient population.

Our results also showed increased BALP and BGP levels in the Repair group when compared to the Non-repair group six months post-operation. Both BALP and BGP are commonly used indicators for clinical evaluation of bone metabolism.^20^ Increased BALP levels can indicate an increase in osteogenic activity, while BGP can reflect the osteogenic ability of osteoblasts. The higher the bone turnover rate, the higher the BGP level.^20^,^21^ Some studies have found that ORIF can significantly improve BALP, BGP, Type-I collagen amino-terminal extender peptide (P1NP) and bone resorption marker β- Type-I collagen carboxyl-terminal peptide (β-CTx) levels in patients with ankle fracture combined with triangular ligament injury.^4^,^20^

It is suspected that repairing the triangular ligament can improve the blood supply of local bone tissue in patients with ankle fractures, promoting the formation of new bone in patients, thereby improving the activity and osteogenic ability of osteoblasts, and significantly improving their bone metabolism.^22^,^23^ However, this study did not monitor P1NP and β- CTx levels, and the observation and evaluation of bone metabolism indicators in patients were not comprehensive enough to confirm this conclusion. Despite this, the study investigated the combination of SA and ORIF from the aspects of overall clinical efficacy, ankle function, pain assessment, and bone metabolism, and showed a favorable outcome.

### Limitation of the study

This was a single-center retrospective analysis, with a relatively short follow-up time of only six months post-operation. The short follow-up time makes it difficult to evaluate the long-term impact of triangular ligament repair. Future research should include a larger sample size and longer follow-up time to further explore the application value of this combined approach to improve the reliability of the research results.

## CONCLUSION

Compared with simple ORIF, SA repair combined with ORIF can significantly improve the efficacy of patients, relieve postoperative pain, and improve ankle function and bone metabolism.

### Authors’ contributions:

**LZ:** Conceived and designed the study.

**XK, GH, YZ** and **LZ:** Collected the data and performed the analysis.

**LZ:** Was involved in the writing of the manuscript and is responsible for the integrity of the study.

All authors have read and approved the final manuscript.
